# Supplementary material for: Splice-Junction-Based Mapping of Alternative Isoforms in the Human Proteome
Source: Cell Rep. Author manuscript; Available in PMC 2020 Jan 15. (PMC6961840; doi:10.1016/j.celrep.2019.11.026)
Supplement: 2 [file NIHMS1546469-supplement-2.zip › DF1/PXD009737/TestisLysC/TestisLysC_13_EPB41L3_RLSTSPVRLAARQEDAPMIEPLVPEEVSSQK.pdf]

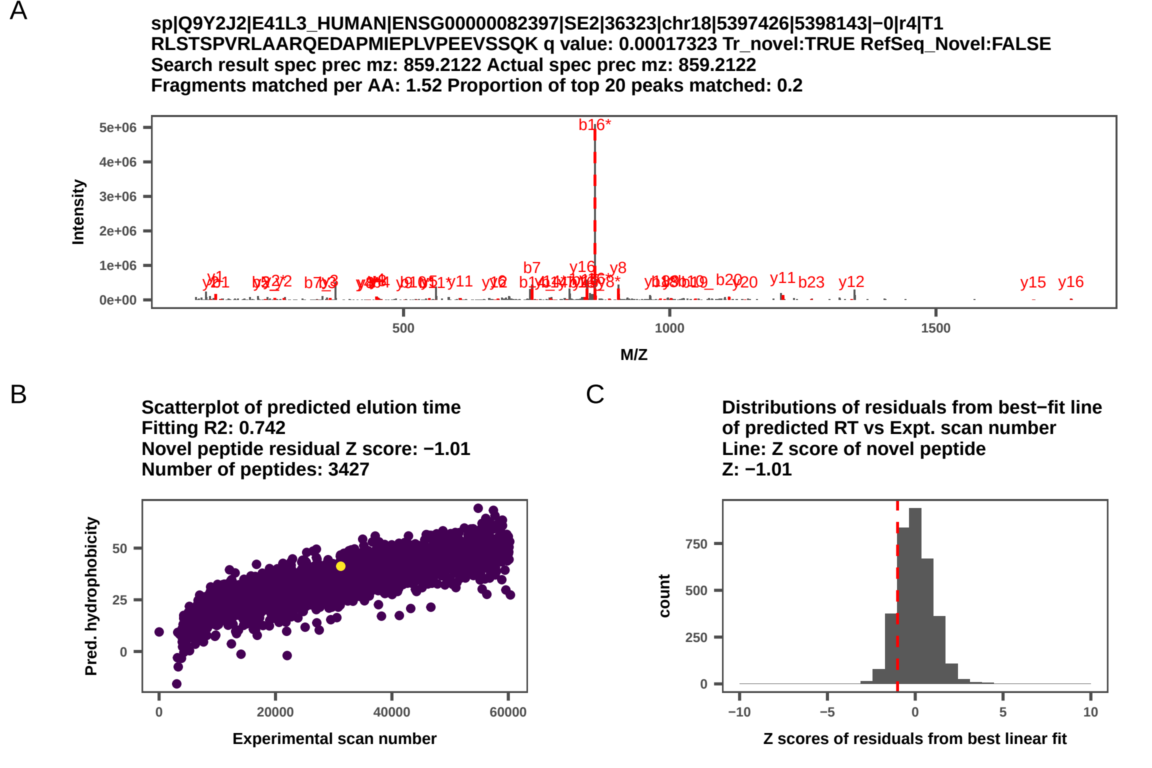
A
sp|Q9Y2J2|E41L3_HUMAN|ENSG00000082397|SE2|36323|chr18|5397426|5398143|−0|r4|T1
RLSTSPVRLAARQEDAPMIEPLVPEEVSSQK q value: 0.00017323 Tr_novel:TRUE RefSeq_Novel:FALSE
Search result spec prec mz: 859.2122 Actual spec prec mz: 859.2122
Fragments matched per AA: 1.52 Proportion of top 20 peaks matched: 0.2
Intensity
5e+06
4e+06
3e+06
2e+06
1e+06
0e+00
b16*
b7
y16
y8
y11
y11
b23
y12
y15
y16
y20
500
1000
1500
M/Z
B
Scatterplot of predicted elution time
Fitting R2: 0.742
Novel peptide residual Z score: −1.01
Number of peptides: 3427
Pred. hydrophobicity
50
25
0
0
20000
40000
60000
Experimental scan number
C
Distributions of residuals from best−fit line
of predicted RT vs Expt. scan number
Line: Z score of novel peptide
Z: −1.01
count
750
500
250
0
−10
−5
0
5
10
Z scores of residuals from best linear fit
